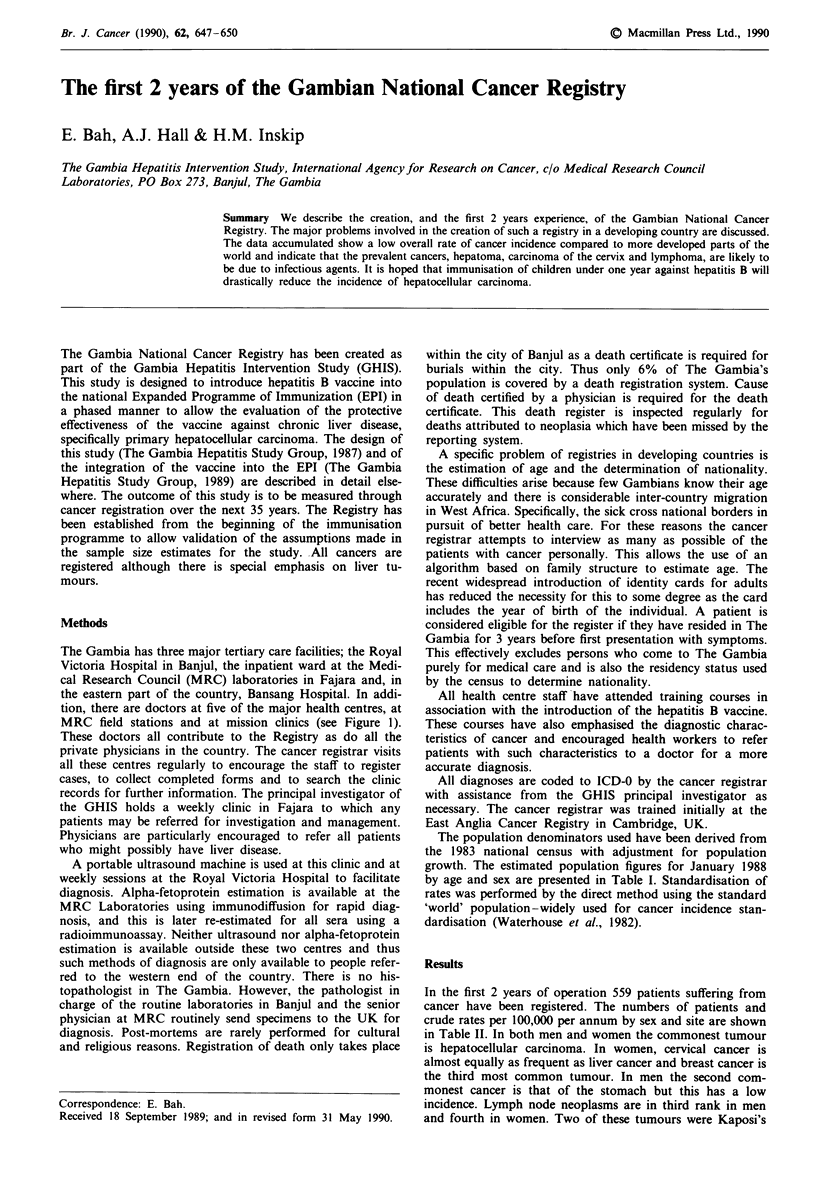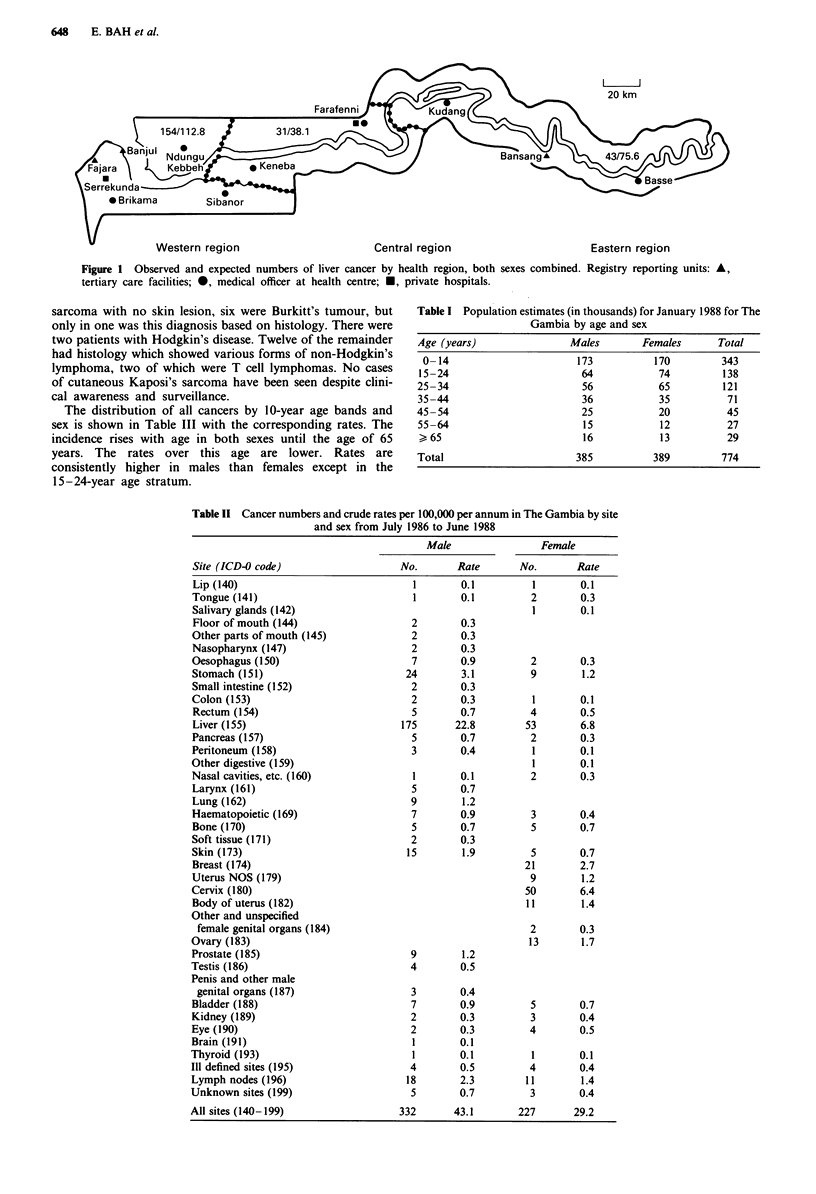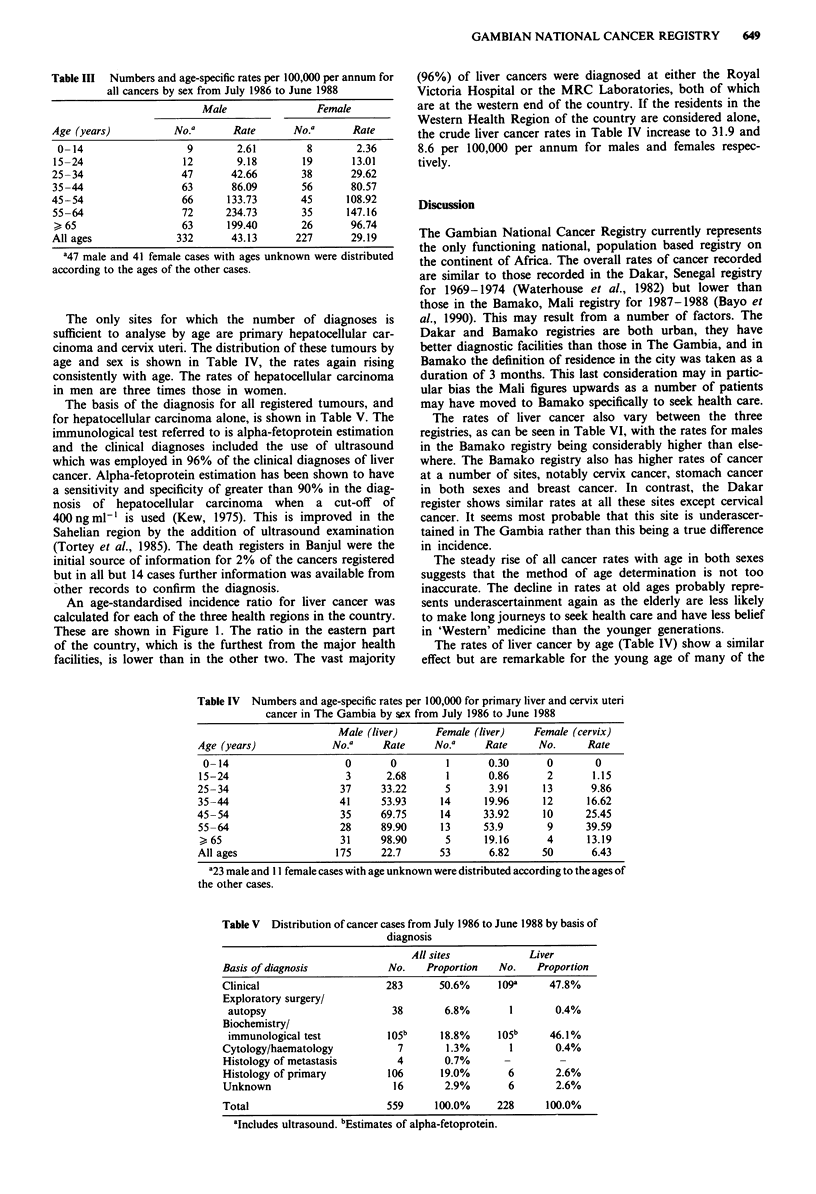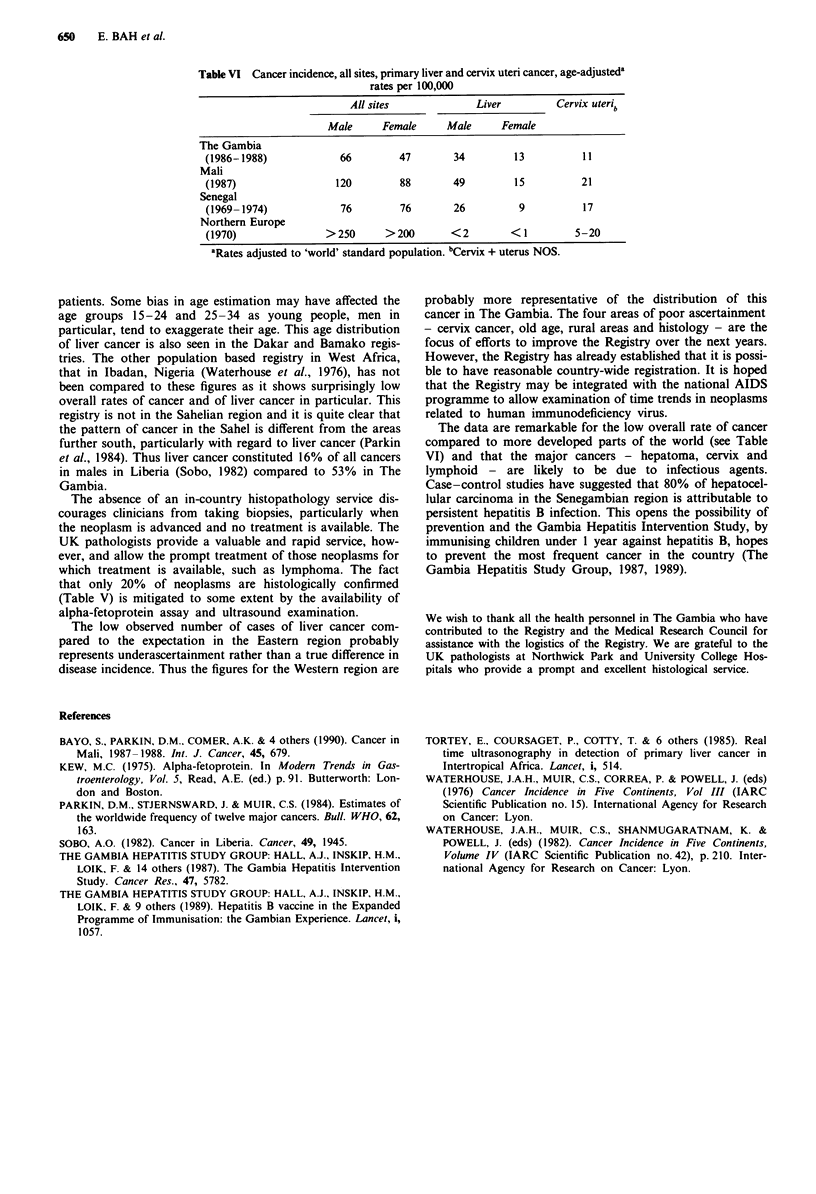# The first 2 years of the Gambian National Cancer Registry.

**DOI:** 10.1038/bjc.1990.347

**Published:** 1990-10

**Authors:** E. Bah, A. J. Hall, H. M. Inskip

**Affiliations:** Gambia Hepatitis Intervention Study, International Agency for Research on Cancer, Banjul.

## Abstract

We describe the creation, and the first 2 years experience, of the Gambian National Cancer Registry. The major problems involved in the creation of such a registry in a developing country are discussed. The data accumulated show a low overall rate of cancer incidence compared to more developed parts of the world and indicate that the prevalent cancers, hepatoma, carcinoma of the cervix and lymphoma, are likely to be due to infectious agents. It is hoped that immunisation of children under one year against hepatitis B will drastically reduce the incidence of hepatocellular carcinoma.


*Br. J. Cancer* (1990), **62**, 647–650 © Macmillan Press Ltd., 1990

### The first 2 years of the Gambian National Cancer Registry

E. Bah, A.J. Hall & H.M. Inskip

*The Gambia Hepatitis Intervention Study, International Agency for Research on Cancer, c/o Medical Research Council Laboratories, PO Box 273, Banjul, The Gambia*

**Summary** We describe the creation, and the first 2 years experience, of the Gambian National Cancer Registry. The major problems involved in the creation of such a registry in a developing country are discussed. The data accumulated show a low overall rate of cancer incidence compared to more developed parts of the world and indicate that the prevalent cancers, hepatoma, carcinoma of the cervix and lymphoma, are likely to be due to infectious agents. It is hoped that immunisation of children under one year against hepatitis B will drastically reduce the incidence of hepatocellular carcinoma.

The Gambia National Cancer Registry has been created as part of the Gambia Hepatitis Intervention Study (GHIS). This study is designed to introduce hepatitis B vaccine into the national Expanded Programme of Immunization (EPI) in a phased manner to allow the evaluation of the protective effectiveness of the vaccine against chronic liver disease, specifically primary hepatocellular carcinoma. The design of this study (The Gambia Hepatitis Study Group, 1987) and of the integration of the vaccine into the EPI (The Gambia Hepatitis Study Group, 1989) are described in detail elsewhere. The outcome of this study is to be measured through cancer registration over the next 35 years. The Registry has been established from the beginning of the immunisation programme to allow validation of the assumptions made in the sample size estimates for the study. All cancers are registered although there is special emphasis on liver tumours.

### Methods

The Gambia has three major tertiary care facilities; the Royal Victoria Hospital in Banjul, the inpatient ward at the Medical Research Council (MRC) laboratories in Fajara and, in the eastern part of the country, Bansang Hospital. In addition, there are doctors at five of the major health centres, at MRC field stations and at mission clinics (see Figure 1). These doctors all contribute to the Registry as do all the private physicians in the country. The cancer registrar visits all these centres regularly to encourage the staff to register cases, to collect completed forms and to search the clinic records for further information. The principal investigator of the GHIS holds a weekly clinic in Fajara to which any patients may be referred for investigation and management. Physicians are particularly encouraged to refer all patients who might possibly have liver disease.

A portable ultrasound machine is used at this clinic and at weekly sessions at the Royal Victoria Hospital to facilitate diagnosis. Alpha-fetoprotein estimation is available at the MRC Laboratories using immunodiffusion for rapid diagnosis, and this is later re-estimated for all sera using a radioimmunoassay. Neither ultrasound nor alpha-fetoprotein estimation is available outside these two centres and thus such methods of diagnosis are only available to people referred to the western end of the country. There is no histopathologist in The Gambia. However, the pathologist in charge of the routine laboratories in Banjul and the senior physician at MRC routinely send specimens to the UK for diagnosis. Post-mortems are rarely performed for cultural and religious reasons. Registration of death only takes place within the city of Banjul as a death certificate is required for burials within the city. Thus only 6% of The Gambia's population is covered by a death registration system. Cause of death certified by a physician is required for the death certificate. This death register is inspected regularly for deaths attributed to neoplasia which have been missed by the reporting system.

A specific problem of registries in developing countries is the estimation of age and the determination of nationality. These difficulties arise because few Gambians know their age accurately and there is considerable inter-country migration in West Africa. Specifically, the sick cross national borders in pursuit of better health care. For these reasons the cancer registrar attempts to interview as many as possible of the patients with cancer personally. This allows the use of an algorithm based on family structure to estimate age. The recent widespread introduction of identity cards for adults has reduced the necessity for this to some degree as the card includes the year of birth of the individual. A patient is considered eligible for the register if they have resided in The Gambia for 3 years before first presentation with symptoms. This effectively excludes persons who come to The Gambia purely for medical care and is also the residency status used by the census to determine nationality.

All health centre staff have attended training courses in association with the introduction of the hepatitis B vaccine. These courses have also emphasised the diagnostic characteristics of cancer and encouraged health workers to refer patients with such characteristics to a doctor for a more accurate diagnosis.

All diagnoses are coded to ICD-0 by the cancer registrar with assistance from the GHIS principal investigator as necessary. The cancer registrar was trained initially at the East Anglia Cancer Registry in Cambridge, UK.

The population denominators used have been derived from the 1983 national census with adjustment for population growth. The estimated population figures for January 1988 by age and sex are presented in Table I. Standardisation of rates was performed by the direct method using the standard 'world' population–widely used for cancer incidence standardisation (Waterhouse *et al.*, 1982).

### Results

In the first 2 years of operation 559 patients suffering from cancer have been registered. The numbers of patients and crude rates per 100,000 per annum by sex and site are shown in Table II. In both men and women the commonest tumour is hepatocellular carcinoma. In women, cervical cancer is almost equally as frequent as liver cancer and breast cancer is the third most common tumour. In men the second commonest cancer is that of the stomach but this has a low incidence. Lymph node neoplasms are in third rank in men and fourth in women. Two of these tumours were Kaposi's

Correspondence: E. Bah.
Received 18 September 1989; and in revised form 31 May 1990.

**Figure 1** Observed and expected numbers of liver cancer by health region, both sexes combined. Registry reporting units: ▲, tertiary care facilities; ●, medical officer at health centre; ■, private hospitals.

sarcoma with no skin lesion, six were Burkitt's tumour, but only in one was this diagnosis based on histology. There were two patients with Hodgkin's disease. Twelve of the remainder had histology which showed various forms of non-Hodgkin's lymphoma, two of which were T cell lymphomas. No cases of cutaneous Kaposi's sarcoma have been seen despite clinical awareness and surveillance.

The distribution of all cancers by 10-year age bands and sex is shown in Table III with the corresponding rates. The incidence rises with age in both sexes until the age of 65 years. The rates over this age are lower. Rates are consistently higher in males than females except in the 15–24-year age stratum.

**Table I** Population estimates (in thousands) for January 1988 for The Gambia by age and sex

| *Age (years)* | *Males* | *Females* | *Total* |
|---|---|---|---|
| 0–14 | 173 | 170 | 343 |
| 15–24 | 64 | 74 | 138 |
| 25–34 | 56 | 65 | 121 |
| 35–44 | 36 | 35 | 71 |
| 45–54 | 25 | 20 | 45 |
| 55–64 | 15 | 12 | 27 |
| ⩾65 | 16 | 13 | 29 |
| Total | 385 | 389 | 774 |

**Table II** Cancer numbers and crude rates per 100,000 per annum in The Gambia by site and sex from July 1986 to June 1988

| | *Male* | | *Female* | |
|---|---|---|---|---|
| *Site (ICD-0 code)* | *No.* | *Rate* | *No.* | *Rate* |
| Lip (140) | 1 | 0.1 | 1 | 0.1 |
| Tongue (141) | 1 | 0.1 | 2 | 0.3 |
| Salivary glands (142) | | | 1 | 0.1 |
| Floor of mouth (144) | 2 | 0.3 | | |
| Other parts of mouth (145) | 2 | 0.3 | | |
| Nasopharynx (147) | 2 | 0.3 | | |
| Oesophagus (150) | 7 | 0.9 | 2 | 0.3 |
| Stomach (151) | 24 | 3.1 | 9 | 1.2 |
| Small intestine (152) | 2 | 0.3 | | |
| Colon (153) | 2 | 0.3 | 1 | 0.1 |
| Rectum (154) | 5 | 0.7 | 4 | 0.5 |
| Liver (155) | 175 | 22.8 | 53 | 6.8 |
| Pancreas (157) | 5 | 0.7 | 2 | 0.3 |
| Peritoneum (158) | 3 | 0.4 | 1 | 0.1 |
| Other digestive (159) | | | 1 | 0.1 |
| Nasal cavities, etc. (160) | 1 | 0.1 | 2 | 0.3 |
| Larynx (161) | 5 | 0.7 | | |
| Lung (162) | 9 | 1.2 | | |
| Haematopoietic (169) | 7 | 0.9 | 3 | 0.4 |
| Bone (170) | 5 | 0.7 | 5 | 0.7 |
| Soft tissue (171) | 2 | 0.3 | | |
| Skin (173) | 15 | 1.9 | 5 | 0.7 |
| Breast (174) | | | 21 | 2.7 |
| Uterus NOS (179) | | | 9 | 1.2 |
| Cervix (180) | | | 50 | 6.4 |
| Body of uterus (182) | | | 11 | 1.4 |
| Other and unspecified female genital organs (184) | | | 2 | 0.3 |
| Ovary (183) | | | 13 | 1.7 |
| Prostate (185) | 9 | 1.2 | | |
| Testis (186) | 4 | 0.5 | | |
| Penis and other male genital organs (187) | 3 | 0.4 | | |
| Bladder (188) | 7 | 0.9 | 5 | 0.7 |
| Kidney (189) | 2 | 0.3 | 3 | 0.4 |
| Eye (190) | 2 | 0.3 | 4 | 0.5 |
| Brain (191) | 1 | 0.1 | | |
| Thyroid (193) | 1 | 0.1 | 1 | 0.1 |
| Ill defined sites (195) | 4 | 0.5 | 4 | 0.4 |
| Lymph nodes (196) | 18 | 2.3 | 11 | 1.4 |
| Unknown sites (199) | 5 | 0.7 | 3 | 0.4 |
| All sites (140–199) | 332 | 43.1 | 227 | 29.2 |

**Table III** Numbers and age-specific rates per 100,000 per annum for all cancers by sex from July 1986 to June 1988

| | *Male* | | *Female* | |
|---|---|---|---|---|
| *Age (years)* | *No.*[a] | *Rate* | *No.*[a] | *Rate* |
| 0–14 | 9 | 2.61 | 8 | 2.36 |
| 15–24 | 12 | 9.18 | 19 | 13.01 |
| 25–34 | 47 | 42.66 | 38 | 29.62 |
| 35–44 | 63 | 86.09 | 56 | 80.57 |
| 45–54 | 66 | 133.73 | 45 | 108.92 |
| 55–64 | 72 | 234.73 | 35 | 147.16 |
| ⩾65 | 63 | 199.40 | 26 | 96.74 |
| All ages | 332 | 43.13 | 227 | 29.19 |

[a]47 male and 41 female cases with ages unknown were distributed according to the ages of the other cases.

The only sites for which the number of diagnoses is sufficient to analyse by age are primary hepatocellular carcinoma and cervix uteri. The distribution of these tumours by age and sex is shown in Table IV, the rates again rising consistently with age. The rates of hepatocellular carcinoma in men are three times those in women.

The basis of the diagnosis for all registered tumours, and for hepatocellular carcinoma alone, is shown in Table V. The immunological test referred to is alpha-fetoprotein estimation and the clinical diagnoses included the use of ultrasound which was employed in 96% of the clinical diagnoses of liver cancer. Alpha-fetoprotein estimation has been shown to have a sensitivity and specificity of greater than 90% in the diagnosis of hepatocellular carcinoma when a cut-off of 400 $\text{ng ml}^{-1}$ is used (Kew, 1975). This is improved in the Sahelian region by the addition of ultrasound examination (Tortey *et al.*, 1985). The death registers in Banjul were the initial source of information for 2% of the cancers registered but in all but 14 cases further information was available from other records to confirm the diagnosis.

An age-standardised incidence ratio for liver cancer was calculated for each of the three health regions in the country. These are shown in Figure 1. The ratio in the eastern part of the country, which is the furthest from the major health facilities, is lower than in the other two. The vast majority (96%) of liver cancers were diagnosed at either the Royal Victoria Hospital or the MRC Laboratories, both of which are at the western end of the country. If the residents in the Western Health Region of the country are considered alone, the crude liver cancer rates in Table IV increase to 31.9 and 8.6 per 100,000 per annum for males and females respectively.

### Discussion

The Gambian National Cancer Registry currently represents the only functioning national, population based registry on the continent of Africa. The overall rates of cancer recorded are similar to those recorded in the Dakar, Senegal registry for 1969–1974 (Waterhouse *et al.*, 1982) but lower than those in the Bamako, Mali registry for 1987–1988 (Bayo *et al.*, 1990). This may result from a number of factors. The Dakar and Bamako registries are both urban, they have better diagnostic facilities than those in The Gambia, and in Bamako the definition of residence in the city was taken as a duration of 3 months. This last consideration may in particular bias the Mali figures upwards as a number of patients may have moved to Bamako specifically to seek health care.

The rates of liver cancer also vary between the three registries, as can be seen in Table VI, with the rates for males in the Bamako registry being considerably higher than elsewhere. The Bamako registry also has higher rates of cancer at a number of sites, notably cervix cancer, stomach cancer in both sexes and breast cancer. In contrast, the Dakar register shows similar rates at all these sites except cervical cancer. It seems most probable that this site is underascertained in The Gambia rather than this being a true difference in incidence.

The steady rise of all cancer rates with age in both sexes suggests that the method of age determination is not too inaccurate. The decline in rates at old ages probably represents underascertainment again as the elderly are less likely to make long journeys to seek health care and have less belief in 'Western' medicine than the younger generations.

The rates of liver cancer by age (Table IV) show a similar effect but are remarkable for the young age of many of the

**Table IV** Numbers and age-specific rates per 100,000 for primary liver and cervix uteri cancer in The Gambia by sex from July 1986 to June 1988

| | *Male (liver)* | | *Female (liver)* | | *Female (cervix)* | |
|---|---|---|---|---|---|---|
| *Age (years)* | *No.*[a] | *Rate* | *No.*[a] | *Rate* | *No.* | *Rate* |
| 0–14 | 0 | 0 | 1 | 0.30 | 0 | 0 |
| 15–24 | 3 | 2.68 | 1 | 0.86 | 2 | 1.15 |
| 25–34 | 37 | 33.22 | 5 | 3.91 | 13 | 9.86 |
| 35–44 | 41 | 53.93 | 14 | 19.96 | 12 | 16.62 |
| 45–54 | 35 | 69.75 | 14 | 33.92 | 10 | 25.45 |
| 55–64 | 28 | 89.90 | 13 | 53.9 | 9 | 39.59 |
| ⩾65 | 31 | 98.90 | 5 | 19.16 | 4 | 13.19 |
| All ages | 175 | 22.7 | 53 | 6.82 | 50 | 6.43 |

[a]23 male and 11 female cases with age unknown were distributed according to the ages of the other cases.

**Table V** Distribution of cancer cases from July 1986 to June 1988 by basis of diagnosis

| | *All sites* | | *Liver* | |
|---|---|---|---|---|
| *Basis of diagnosis* | *No.* | *Proportion* | *No.* | *Proportion* |
| Clinical | 283 | 50.6% | 109[a] | 47.8% |
| Exploratory surgery/ autopsy | 38 | 6.8% | 1 | 0.4% |
| Biochemistry/ immunological test | 105[b] | 18.8% | 105[b] | 46.1% |
| Cytology/haematology | 7 | 1.3% | 1 | 0.4% |
| Histology of metastasis | 4 | 0.7% | – | – |
| Histology of primary | 106 | 19.0% | 6 | 2.6% |
| Unknown | 16 | 2.9% | 6 | 2.6% |
| Total | 559 | 100.0% | 228 | 100.0% |

[a]Includes ultrasound. [b]Estimates of alpha-fetoprotein.

**Table VI** Cancer incidence, all sites, primary liver and cervix uteri cancer, age-adjusted[a] rates per 100,000

| | *All sites* | | *Liver* | | *Cervix uteri*[b] |
|---|---|---|---|---|---|
| | *Male* | *Female* | *Male* | *Female* | |
| The Gambia (1986–1988) | 66 | 47 | 34 | 13 | 11 |
| Mali (1987) | 120 | 88 | 49 | 15 | 21 |
| Senegal (1969–1974) | 76 | 76 | 26 | 9 | 17 |
| Northern Europe (1970) | >250 | >200 | <2 | <1 | 5–20 |

[a]Rates adjusted to 'world' standard population. [b]Cervix + uterus NOS.

patients. Some bias in age estimation may have affected the age groups 15–24 and 25–34 as young people, men in particular, tend to exaggerate their age. This age distribution of liver cancer is also seen in the Dakar and Bamako registries. The other population based registry in West Africa, that in Ibadan, Nigeria (Waterhouse *et al.*, 1976), has not been compared to these figures as it shows surprisingly low overall rates of cancer and of liver cancer in particular. This registry is not in the Sahelian region and it is quite clear that the pattern of cancer in the Sahel is different from the areas further south, particularly with regard to liver cancer (Parkin *et al.*, 1984). Thus liver cancer constituted 16% of all cancers in males in Liberia (Sobo, 1982) compared to 53% in The Gambia.

The absence of an in-country histopathology service discourages clinicians from taking biopsies, particularly when the neoplasm is advanced and no treatment is available. The UK pathologists provide a valuable and rapid service, however, and allow the prompt treatment of those neoplasms for which treatment is available, such as lymphoma. The fact that only 20% of neoplasms are histologically confirmed (Table V) is mitigated to some extent by the availability of alpha-fetoprotein assay and ultrasound examination.

The low observed number of cases of liver cancer compared to the expectation in the Eastern region probably represents underascertainment rather than a true difference in disease incidence. Thus the figures for the Western region are probably more representative of the distribution of this cancer in The Gambia. The four areas of poor ascertainment – cervix cancer, old age, rural areas and histology – are the focus of efforts to improve the Registry over the next years. However, the Registry has already established that it is possible to have reasonable country-wide registration. It is hoped that the Registry may be integrated with the national AIDS programme to allow examination of time trends in neoplasms related to human immunodeficiency virus.

The data are remarkable for the low overall rate of cancer compared to more developed parts of the world (see Table VI) and that the major cancers – hepatoma, cervix and lymphoid – are likely to be due to infectious agents. Case–control studies have suggested that 80% of hepatocellular carcinoma in the Senegambian region is attributable to persistent hepatitis B infection. This opens the possibility of prevention and the Gambia Hepatitis Intervention Study, by immunising children under 1 year against hepatitis B, hopes to prevent the most frequent cancer in the country (The Gambia Hepatitis Study Group, 1987, 1989).

We wish to thank all the health personnel in The Gambia who have contributed to the Registry and the Medical Research Council for assistance with the logistics of the Registry. We are grateful to the UK pathologists at Northwick Park and University College Hospitals who provide a prompt and excellent histological service.

### References

BAYO, S., PARKIN, D.M., COMER, A.K. & 4 others (1990). Cancer in Mali, 1987–1988. *Int. J. Cancer*, **45**, 679.

KEW, M.C. (1975). Alpha-fetoprotein. In *Modern Trends in Gastroenterology, Vol. 5*, Read, A.E. (ed.) p. 91. Butterworth: London and Boston.

PARKIN, D.M., STJERNSWARD, J. & MUIR, C.S. (1984). Estimates of the worldwide frequency of twelve major cancers. *Bull. WHO*, **62**, 163.

SOBO, A.O. (1982). Cancer in Liberia. *Cancer*, **49**, 1945.

THE GAMBIA HEPATITIS STUDY GROUP: HALL, A.J., INSKIP, H.M., LOIK, F. & 14 others (1987). The Gambia Hepatitis Intervention Study. *Cancer Res.*, **47**, 5782.

THE GAMBIA HEPATITIS STUDY GROUP: HALL, A.J., INSKIP, H.M., LOIK, F. & 9 others (1989). Hepatitis B vaccine in the Expanded Programme of Immunisation: the Gambian Experience. *Lancet*, **i**, 1057.

TORTEY, E., COURSAGET, P., COTTY, T. & 6 others (1985). Real time ultrasonography in detection of primary liver cancer in Intertropical Africa. *Lancet*, **i**, 514.

WATERHOUSE, J.A.H., MUIR, C.S., CORREA, P. & POWELL, J. (eds) (1976) *Cancer Incidence in Five Continents, Vol III* (IARC Scientific Publication no. 15). International Agency for Research on Cancer: Lyon.

WATERHOUSE, J.A.H., MUIR, C.S., SHANMUGARATNAM, K. & POWELL, J. (eds) (1982). *Cancer Incidence in Five Continents, Volume IV* (IARC Scientific Publication no. 42), p. 210. International Agency for Research on Cancer: Lyon.